# Non-Communicable Disease Mortality and Risk Factors in Formal and Informal Neighborhoods, Ouagadougou, Burkina Faso: Evidence from a Health and Demographic Surveillance System

**DOI:** 10.1371/journal.pone.0113780

**Published:** 2014-12-10

**Authors:** Clémentine Rossier, Abdramane Bassiahi Soura, Géraldine Duthé, Sally Findley

**Affiliations:** 1 Institute of Demographic and Life Course Studies, University of Geneva, Geneva, Switzerland; 2 Institut National d’Etudes Démographiques, Paris, France; 3 Institut Supérieur des Sciences de la Population, University of Ouagadougou, Ouagadougou, Burkina Faso; 4 Mailman School of Public Health, Columbia University, New York, New York, United States of America; Leibniz Institute for Prevention Research and Epidemiology (BIPS), Germany

## Abstract

The expected growth in NCDs in cities is one of the most important health challenges of the coming decades in Sub-Saharan countries. This paper aims to fill the gap in our understanding of socio-economic differentials in NCD mortality and risk in low and middle income neighborhoods in urban Africa. We use data collected in the Ouagadougou Health and Demographic Surveillance System. 409 deaths were recorded between 2009–2011 among 20,836 individuals aged 35 years and older; verbal autopsies and the InterVA program were used to determine the probable cause of death. A random survey asked in 2011 1,039 adults aged 35 and over about tobacco use, heavy alcohol consumption, lack of physical activity and measured their weight, height, and blood pressure. These data reveal a high level of premature mortality due to NCDs in all neighborhoods: NCD mortality increases substantially by age 50. NCD mortality is greater in formal neighborhoods, while adult communicable disease mortality remains high, especially in informal neighborhoods. There is a high prevalence of risk factors for NCDs in the studied neighborhoods, with over one-fourth of the adults being overweight and over one-fourth having hypertension. Better-off residents are more prone to physical inactivity and excessive weight, while vulnerable populations such as widows/divorced individuals and migrants suffer more from higher blood pressure. Females have a significantly lower risk of being smokers or heavy drinkers, while they are more likely to be physically inactive or overweight, especially when married. Muslim individuals are less likely to be smokers or heavy drinkers, but have a higher blood pressure. Everything else being constant, individuals living in formal neighborhoods are more often overweight. The data presented make clear the pressing need to develop effective programs to reduce NCD risk across all types of neighborhoods in African cities, and suggest several entry points for community-based prevention programs.

## Introduction

Non-communicable diseases (NCDs) now account for more than half of the global burden of disease and this share is expected to continue to increase in the coming decades [1]. The greatest increase is expected in Africa, where deaths from NCDs are expected to exceed deaths from communicable diseases by 2030 [2], [3]. This shift in the balance of causes of death has several roots. As child mortality decreases in the poorest countries, more individuals survive to adulthood, when the risk of NCD death is greater [4]. At the same time, the on-going fertility decline further changes the age composition, toward more adults than children. Overlaying these demographic trends, however, are the important economic, social, and cultural transformations in low income countries which propel more individuals to adopt less healthy habits. More sedentary lifestyles, unhealthier diets, and increases in tobacco and alcohol intake, lead to higher levels of blood pressure, cholesterol and obesity, and to more NCDs [2], [5].

These changes are magnified among the burgeoning urban populations in low income countries. Data from seven Sub Saharan African countries show that 31% of the adults were obese in cities, compared to 14% among the rural population [6], [7]. While the level of hypertension varies greatly between studies [8], its prevalence in Sub-Saharan Africa is also consistently higher in urban areas [8], [9]. At the same time, Sub-Saharan Africa is the region with the highest projected increases in urban population in the coming decades, from 36.3% in 2010 to 56.5% in 2030 [10]. The expected growth in NCDs in cities is one of the most important health challenges of the coming decades in Sub-Saharan countries.

In other regions of the world, the poor and slum dwellers are more likely to be obese and/or have cardiovascular diseases [5]–[7]; if this holds true for sub-Saharan Africa the expected rapid growth rate of Africa’s urban poor [10], [11] is likely to be accompanied by massive increases in NCD prevalence in Africa’s low-income neighborhoods. However, NCDs also touch all social classes and all types of neighborhoods [1], [4], [6], and could concern, in the context of urban Africa, wealthier as much as poorer residents. A sound knowledge based on socio-economic and contextual differentials in NCDs and their risk factors in urban Sub-Saharan Africa is needed to develop well-targeted NCD prevention and management strategies for African cities. This paper aims to fill the gap in our understanding of socio-economic differentials in NCD risk in low and middle income neighborhoods in urban Africa. Using data from Ouagadougou, capital of Burkina Faso, it documents differences in NCD mortality among adults aged 35 and over in formal versus informal neighborhoods. It also documents prevalence of behavioral risk factors of NCDs in these neighborhoods.

## Materials and Methods

### Context and population

Data come from the Ouagadougou Health and Demographic Surveillance System, which was established in 2008 to monitor population and health trends in five neighborhoods located at the periphery of Ouagadougou, Burkina Faso [12]. Two of these neighborhoods (Kilwin and Tanghin) are formal, while the three others (Nonghin, Polesgo and Nioko 2) are unplanned and informal peri-urban settlements (i.e. slums). Their total populations are 40,775 and 43,073, respectively. Formal areas are organized according to modern ownership rights, with each housing plot being registered is the city’s land registry and having access to city electricity, water and other services. Informal areas are typically located at the periphery of the city, on village lands bordering the city, and are still ruled by customary land ownership; new comers informally buy plots from villagers. Their ownership rights are semi-formal, in the sense that the city gives priority to local residents when zoning informal settlements, which it has been doing periodically. The only public services available in informal settlements are those existing in the old villages [13], [14].

Detailed demographic, socio-economic and health data have been collected through regular updates on all households within the surveillance site. Table 1 highlights the differences between the formal and informal neighborhoods. Households in informal areas are smaller; there are more children below age 5 and fewer adults over age 50. Compared to adults in the formal areas, those in informal areas are more likely to be poor and to have never gone to formal school, but socio-economic status in the neighborhoods remains relatively mixed. These characteristics are explained by the fact that informal neighborhoods attract single men or young nuclear families with small children in search of affordable housing. Rural-urban migrants are numerous in all peripheral neighborhoods (formal and informal), more so in informal areas; however, most individuals have resided elsewhere in the city before moving to their current homes. Households in informal areas have no access to private piped water or organized garbage collection. They face more difficult access to public services in general. Informal areas are not equipped with secondary schools nor public health centers, except one dispensary in the old village of Polesgo; residents have to use the facilities located in formal areas.

**Table 1:** Demographic, socio-economic and housing characteristics of the population living in the Ouaga HDSS, by type of neighborhood (formal and informal), 2011.

		Formal	Informal	All
Total population	Age			
	0–4	11.6%	19.3%	15.6%
	5–14	22.5%	23.6%	23.1%
	15–34	43.0%	38.3%	40.6%
	35–49	13.8%	13.5%	13.6%
	50–64	6.7%	3.9%	5.2%
	65 and over	2.4%	1.4%	1.9%
	**N**	40,775	43,073	83,848
**Individuals aged 15+**	**Level of education**			
	None	32.3%	53.6%	42.5%
	Primary	24.8%	27.0%	25.9%
	Secondary	42.9%	19.4%	31.6%
	**Standard of living** *			
	Poor	22.9%	61.7%	39.9%
	Middle	55.2%	37.5%	47.4%
	Rich	21.9%	0.8%	12.%
	**Place of birth**			
	Ouagadougou	39.7%	21.2%	30.8%
	Other	60.3%	78.8%	69.2%
	**Last residence**			
	Ouagadougou	68.9%	75.3%	72.0%
	Other	31.1%	24.7%	28.0%
	**N**	24,557	22,496	47,053
**Households**	**Average size**	5.8	3.8	4.5
	**Standard of living**			
	Poor	18.7%	52.7%	39.7%
	Middle	59.7%	46.3%	51.4%
	Rich	21.6%	1.0%	8.9%
	**No private piped water**	44.9%	96.7%	76.9%
	**Organized garbage collection**	53.1%	0.8%	20.7%
	**N**	6,659	10,823	17,482

*Each individuals aged 15 and more was given the standard of living of its household.

### The mortality data

After an initial census conducted between October 2008 and March 2009 in the five neighborhoods, fieldworkers make household visits on average every 7 months to register vital events, mainly births and deaths, migrations and marriages. In case of death, a verbal autopsy (VA) questionnaire is completed with the next of kin to determine the circumstances that led to the death, including history of the illness and the specific symptoms that preceded death. This VA protocol is approved by World Health Organization (WHO) and is used by most members of INDEPTH Network (the International Network for the Demographic Evaluation of Populations and Their Health). Results of the VA are entered into InterVA software (4^th^ version) also approved by WHO to determine the probable cause of death. This program has already been widely tested; while neither InterVA nor diagnoses by medical doctors can be considered as gold standards, InterVA performs at least as well as medical diagnoses [15]–[17].

Data for this analysis cover the time period 2009–2011 and include 409 deaths recorded among 20,836 individuals aged 35 years and older. Of the 409 recorded deaths, 338 had VAs completed (82.6%), and the InterVA4 software was able to assign cause of death for 94.7% of these (the remaining are classified among ill-defined causes). More deaths failed to have a completed VA (20.6%) in informal than formal areas (15.9%). Slightly more deaths failed to have a completed VA (17.4%) among individuals aged 35 years and over compared to all ages (15.1%). We first compare across formal or informal areas the distribution of more detailed causes of deaths, excluding VA not done and indeterminate causes. Then, we calculate cause-specific mortality rates for the formal and informal areas for three broad groups of causes: communicable diseases (including HIV/AIDS, diarrheal diseases, direct obstetric, infectious diseases, malaria, malnutrition, meningitis, neonatal causes, respiratory tract Infection, TB), NCDs (including anemia, asthma, cardio-vascular, chronic obstructive pulmonary disease, diabetes mellitus, liver disease, neoplasms, acute abdomen, epilepsy, other unspecified non communicable diseases) and external causes of death (injuries and violent deaths). We do not display results for external causes, because of the small number of cases (n = 26). We proportionally redistribute the deaths without a VA and the indeterminate causes across the three groups of causes according to the distribution of known cases. We examine differences in mortality rates between age group, sex, educational attainment and household standard of living. While the differences are not statistically significant due to the small number of cases, these analyses help assess the directions of the relations. When looking at totals, we standardize the deaths rates by age to control for age differences among those sampled in the formal and informal areas.

### The Health Survey

We conducted a random household survey from February to June 2010 within the Ouaga HDSS in order to collect detailed information about health care and health care behavior, with a particular focus on NCD risk and prevalence. We first drew a systematic random sample of 1941 households using the Ouaga HDSS database as the sampling frame; in part of the households we interviewed all adults, and in the rest only adults 50 years and over. Altogether, adults in 1699 households were surveyed, out of 1941 sampled households for a response rate of 87.5%, 88.7% in formal areas and 86.6% in informal ones. Almost all non-responses were due to the residents being absent; we registered only a few cases of refusal. We oversampled those age 50 and over, to increase the number of seniors, those most likely to have diagnosed NCDs. In this analysis, we use weights taking into account the stratified sampling strategy and non-response rate. A total of 2351 adults aged 15 and over completed the survey.

The survey included both questionnaires and a short physical examination. The survey was conducted by the Ouaga HDSS fieldworkers. A medical doctor trained them during one week on the questionnaire; another medical doctor trained and supervised the team for the anthropometric measures. Fieldworkers asked adults about the following NCD behavioral risk factors: tobacco use, heavy alcohol consumption, lack of physical activity. We also measured weight, height, and blood pressure. Smokers were defined as currently smoking and for at least one year prior to the survey. For alcohol consumption, the reference period was the last week; following World Health Organization (WHO) standards [18], we defined heavy drinkers as individuals drinking at least 5 standards of alcohol or more (10–12 grams of pure alcohol) one day per week measured over the last week. Adequate exercise was assessed according to WHO standards recommending that adults have 75 minutes of strenuous exercise or 150 minutes of moderate exercise per week [19]. Interviewers asked respondents whether they did any vigorous exercise (such as physical work, bicycling, walking) during the last week, and if yes, how many hours and minutes of exercise on a typical day. Given the mixed nature of this variable (including both moderate and strenuous exercise), we used a 150 minute benchmark, a procedure which underestimates to some extent the number of individuals with an adequate level of exercise. A respondent was classified as overweight if their Body Mass Index (BMI) was 25 or above. Blood pressure was measured by the interviewer using a digital automatic sphygmomanometer (Omron 3) with an appropriate cuff size. Both diastolic and systolic blood pressures were measured three times for all respondents 15 and older at the beginning of the interview after a 5 minutes rest, and then again at the end of each interview; a minimum of 30 seconds was observed between each consecutive measure. We calculated the average of the last two measures, and defined a hypertensive person as someone with a systolic blood pressure of 140 and over and/or a diastolic blood pressure of 90 and over. We compare these various risk factors across formal and informal areas, restricting the analysis to individuals aged 35 years and over (n = 1,039). To understand the differences between formal and informal neighborhoods, we include a series of individual demographic and socio-economic characteristics in the analyses (all drawn from the routine data collection): sex, age and marital status, household’s standard of living, individuals’ educational level, occupational status, ethnicity, religion, and place of birth. For the household’s standard of living, we created a proxy variable that takes into account the presence of durable goods (a refrigerator and a television), as well as the most expensive mode of transport available in the home. Given the rarity of public transportation in Ouagadougou and the spread of the city, households usually own at least a bicycle, a motorcycle or a car. Possession of the most expensive mode of transport discriminates households better than separate counts of bicycles, motorcycles and cars: indeed, the total number of transportation means also depends on the size of the household. The coefficient attached to each good is derived from a principal components analysis (PCA). Based on the scores of the first factor of the PCA (which accounts for 50.4% of the variance), three categories of household were delineated in the HDSS: the poorest, the middle and the wealthiest. The poorest households do not own any of the listed goods, while most of the richest own a refrigerator, a television and a motorbike, and half of them have a car. We also control for individual place of birth (in Ouagadougou versus outside Ouagadougou). Occupational status has three categories: no occupation refers to housewives, retirees and jobless individuals; employees receive a salary from an employer (in the formal or informal sector); independent workers work on their own, usually in the informal sector.

Table 2 displays the demographic and socio-economic characteristics of the health survey respondents aged 35 years and over. Overall, the sample shows a relative balance between the two sexes. It consists predominantly of uneducated individuals, most of whom are in a union, Muslim, Mossi, born outside of Ouagadougou, and whose household is defined as “middle class” or poor. One in two respondents works independently, i.e., as a craftsman, an unskilled worker or a shopkeeper in the informal economy. The elderly are few in the weighted sample; the population of adults is slightly younger in informal settlements.

**Table 2:** Demographic and socio-economic characteristics of individuals aged 35 and more in the sample, by type of neighborhood (formal and informal), Ouagadougou HDSS health survey 2010.

	Formal	Informal	All
**Sex**			
Male	42.9%	56.9%	48.9%
Female	57.1%	43.1%	51.1%
**Age**			
35–49	41.8%	55.2%	47.5%
50–64	41.2%	33.6%	38.1%
65 and more	17.0%	11.2%	14.4%
**Marital status**			
In union	79.1%	78.8%	79.0%
Not in union	20.9%	21.2%	21.0%
**Standard of living**			
Poor	26.4%	63.9%	42.5%
Middle	57.4%	36.1%	48.2%
Rich	16.2%	0.0%	9.3%
**Level of education**			
None	65.0%	78.5%	70.8%
Primary	17.5%	14.7%	16.3%
Secondary or more	17.5%	6.8%	12.9%
**Occupation**			
None	38.8%	30.3%	35.1%
Employee	15.9%	15.8%	15.9%
Independent and others	45.3%	53.9%	49.0%
**Ethnicity**			
Mossi	90.7%	92.7%	91.6%
Other	9.3%	7.3%	8.4%
**Religion**			
Christian	43.1%	43.3%	43.2%
Muslim	56.9%	56.7%	56.8%
**Place of birth**			
Ouagadougou	18.8%	18.9%	18.8%
Other	81.2%	81.1%	81.2%
**N**	**605**	**434**	**1,039**

We examined the bivariate associations between individuals’ socio-demographic characteristics and the risk factors of interest, using the Chi-Square test to distinguish significant associations between categorical variables and risk factors. To understand the observed differences between formal and informal areas, we then performed a series of stepwise logistic regressions on each risk factor, first introducing the place of residence in the regression, and then all the other socioeconomic indicators in turn. Standardized age is used as a continuous variable in the regressions.

### Ethics Statement

The protocols for the health survey and routine HDSS data collection were approved by the Ethics Committee for Health Research of the Ministry of Health of Burkina Faso. All participants provided their written informed consent to participate in this study. Illiterate respondents were asked to find a literate witness, and the informed consent form was read to both the respondent and the witness; if the respondent agreed to participate, he/she was asked for his/her finger print on the consent form, and the witness was asked for his/her signature. These consent procedures were approved by the Ethics Committee for Health Research of the Ministry of Health of Burkina Faso.

## Results

### Cause-specific mortality

In the formal neighborhoods, the NCDs dominate as a cause of death among adults aged 35 and over, accounting for three-fourths of these deaths (Table 3). However, only half of all adult deaths are due to NCDs in informal areas. The most common causes of death after age 35 in formal areas are cardiovascular diseases (35.4% of all deaths), followed by cancers (21.7%), whereas in the informal areas these account for 16.1% and 23.7%, respectively. In the formal neighborhoods, communicable diseases account for only 20.8% of deaths, whereas they account for 40.9% of all deaths in the informal neighborhoods. Respiratory infections and tuberculosis account for most deaths by communicable disease among adults. HIV/AIDS is reported as the cause of death for 4.4% of adult deaths in the study site. While the prevalence of HIV/AIDS in the Ouaga HDSS is not known, according to the 2010 Demographic and Health survey, 2.2% of individuals aged 15 to 49 are HIV positive in Ouagadougou.

**Table 3:** Cause of death distribution among individuals aged 35 years and older by type of neighborhood, Ouaga HDSS, 2009–2011.

	Formal (%)	Informal (%)	Total (%)
**Communicable Diseases**	**20.8**	**40.9**	**26.6**
Respiratory tract infection	5.8	15.1	8.5
Tuberculosis	5.3	9.7	6.6
HIV/AIDS	4.0	5.4	4.4
Malaria	1.8	4.3	2.5
Diarrhoeal Disease	1.8	3.2	2.2
Meningitis	0.9	2.2	1.3
Direct obstetric causes	0.9	1.1	0.9
Malnutrition	0.4	0.0	0.3
**Non-Communicable Diseases**	**73.9**	**50.5**	**67.1**
Cardiovascular diseases	35.4	16.1	29.8
Neoplasms	21.7	23.7	22.3
Other NCDs	10.2	9.7	10.0
Asthma	3.5	1.1	2.8
Diabetes Mellitus	2.6	0.0	1.9
Liver Diseases	0.4	0.0	0.3
**External causes**	**5.3**	**8.6**	**6.3**
Total	100.0	100.0	100.0
Number of deaths	284	125	409

As shown in Table 4, even after controlling for the difference in age structure, overall, NCD mortality is much higher in formal areas, while mortality rates due to communicable diseases are slightly higher in informal areas. NCD mortality rates increase strongly among the older age groups, especially in formal areas. Men have a higher risk of dying than women, regardless of neighborhood or cause of death, but most of these differences are not significant due to the small number of cases. Also, individuals living in richer households have lower mortality rates, for both groups of causes and for both types of neighborhood. While those who have a higher level of formal education have lower NCD mortality, educational attainment does not differentiate communicable disease mortality rates. However, the differences are not always significant due to the small number of cases.

**Table 4:** Mortality rates per 1000 person years (95% CI)**, by age group, sex, educational level, household standard of living and by category of death and neighborhood for individuals aged 35 years and older, Ouaga HDSS, 2009–2011.

	Formal areas		Informal areas	
	Communicable diseases	Non-Communicable Diseases	Communicable diseases	Non-Communicable Diseases
**Age group**				
35–49	**1.4 (0.9–2.2)**	**2.5 (1.8–3.6)**	**1.0 (0.6–1.9)**	**1.3 (0.8–2.2)**
50–64	**2.4 (1.5–4.0)**	**10.1 (7.9–12.9)**	**4.5 (2.7–7.7)**	**6.3 (4.1–9.8)**
65+	**7.5 (4.7–12.1)**	**35.7 (28.7–44.4)**	**13.8 (8.3–22.9)**	**14.4 (8.8–23.5)**
**Sex**				
Male	**2.9 (2.0–4.1)**	**10.4 (8.6–12.5)**	2.9 (1.9–4.2)	3.6 (2.6–5.1)
Female	**1.8 (1.1–2.8)**	**6.0 (4.7–7.6)**	2.5 (1.5–4.2)	2.7 (1.7–4.5)
**Level of education**				
None	2.5 (1.5–4.0)	**11.3 (9.3–13.8)**	3.3 (2.3–4.7)	3.8 (2.7–5.3)
Primary	2.4 (1.1–5.2)	**5.9 (3.8–9.3)**	1.7 (0.7–4.6)	2.7 (1.2–6.0)
Secondary or more	2.4 (1.1–5.0)	4.1 (2.5–6.8)	0.8 (0.1–5.9)	1.7 (0.4–6.9)
**Standard of living**				
Poor	3.3 (2.0–5.6)	10.6 (8.0–14.1)	3.4 (2.3–4.8)	4.1 (3.0–5.7)
Middle	2.3 (1.6–3.4)	**9.0 (7.4–10.9)**	0.3 (1.1–3.6)	**2.1 (1.2–3.7)**
Rich	1.8 (0.9–3.5)	**4.7 (3.1–7.1)**	-n <20	**7.1 (1.0–50.5)**
**All** *	2.3 (1.8–3.1)	8.2 (7.0–9.5)	2.7 (2.0–3.7)	3.3 (2.5–4.3)

Note: Bold indicates significant differences across variable categories.

*Standardized by age **The parentheses indicate a 95% confidence interval around the estimated mortality rates; a difference between rates is significant when the two confidence intervals do not overlap.

### NCD risk factors and associated characteristics

Table 5 shows that neither smoking nor excessive alcohol consumption is common in any of these neighborhoods among adults, although these behaviors are more widespread among certain subgroups. Fewer than one in ten (8.0%) adults age 35 and over are current smokers (Table 5). Smokers are more frequent in informal areas, and are male, younger, married, better educated, and work. Neither religion, nor ethnicity, nor place of birth is associated with significant differences in current smoking rate. Even fewer of the adults age 35 and over in these neighborhoods are heavy drinkers, with a heavy drinking rate estimated at only 5.3%. Neither neighborhood of residence nor socio-economic status is associated with different heavy drinking rates, but drinking does differ by gender, age, and religion, with the highest drinking rates (11.5%) among Christians.

**Table 5:** Prevalence of risk factors for non-communicable diseases by socio-demographic characteristics (%), Ouagadougou HDSS, Health Survey 2010.

	Smoker (% current smoker)	Heavy Drinker (% heavy drinker)	Low Exercise (% inadequate weekly level)	Overweight (% BMI> = 25)	Hypertensive (%)
**Neighborhoo**	p = 0.0141	p = 0.0141	p = 0.0141	p = 0.000	p = 0.0451
Formal	5.8	6.1	60.9	38.0	29.9
Informal	10.9	4.2	44.7	15.7	23.6
**Se**	p = 0.000	p = 0.0956	p = 0.0014	p = 0.000	p = 0.1257
Male	16.0	6.5	48.0	20.7	24.7
Female	0.3	4.2	59.5	35.8	29.5
**Age**	p = 0.0003	p = 0.0000	p = 0.0000	p = 0.0000	p = 0.0000
35–49	11.8	1.3	51.0	34.1	16.1
50–64	5.6	9.3	50.4	26.9	32.2
65 and more	1.9	8.0	72.8	13.5	50.5
**Marital status**	p = 0.0016	p = 0.2673	p = 0.0488	p = 0.0147	p = 0.000
In union	9.7	4.9	60.4	30.4	23.8
Not in union	1.8	6.8	52.1	21.0	39.8
**Standard of living**	p = 0.3895	p = 0.4509	p = 0.0001	p = 0.000	p = 0.6325
Poor	8.8	6.1	46.5	18.0	27.5
Middle	8.1	5.1	53.5	32.1	27.8
Rich	3.8	2.8	74.3	57.1	22.4
**Level of education**	p = 0.005	p = 0.8093	0.1662	p = 0.000	p = 0.0429
None	5.9	5.3	51.8	23.1	29.5
Primary	12.9	6.0	56.4	35.1	24.5
Secondary or more	13.7	4.3	62.0	49.1	17.6
**Occupation**	p = 0.0076	p = 0.4748	p = 0.000	p = 0.9042	p = 0.0006
None	3.8	6.5	67.4	27.4	34.3
Employee	11.9	4.3	49.1	29.1	16.2
Independent	9.7	4.8	45.8	28.9	25.6
**Ethnicity**	p = 0.2879	p = 0.7529	p = 0.1583	p = 0.0083	p = 0.9744
Mossi	8.3	5.4	53.1	27.1	27.1
Other	4.4	4.6	62.4	42.8	27.3
**Religion**	p = 0.0782	p = 0.0000	p = 0.8793	p = 0.5108	p = 0.0137
Christian	10.1	11.5	54.2	29.4	23.1
Muslim	6.4	0.6	53.7	27.5	30.3
**Place of birth**	p = 0.4020	p = 0.7234	p = 0.8846	p = 0.1162	p = 0.0082
Ouagadougou	9.8	5.8	53.3	33.8	18.9
Other	7.6	5.2	54.0	27.2	29.1
**All (n = 1,039)**	8.0	5.3	53.9	28.4	27.2

Note: The first category is always the reference category.

Half (53.9%) of all adults aged 35 years and over do not exercise enough. Those who get insufficient exercise are more likely in the formal neighborhoods and are female, married, older, not working and better off economically. Somewhat less than half (46.5%) of the poorest individuals aged 35 years and over do not get enough exercise, compared to three quarters (74.3%) of the richest individuals.

Over one in four (28.4%) of the adult population aged 35 and over are overweight. While few adults are overweight at the national level, other studies have found comparatively high levels of excessive weight in Ouagadougou [20], [21]. The overweight rate is more than two times higher in the formal (38.0%) than in the informal neighborhoods (15.7%). The propensity to be overweight follows much the same patterns as physical inactivity, with women, better-off economically and more educated more often overweight. In addition to these patterns shared with exercise levels, the non-Mossi and relatively younger adults (aged 35 to 49 versus 50 and over) are more often overweight.

Between one quarter and one-third of all adults (27.2%) have high blood pressure. Another study has found comparable level of hypertension in Ouagadougou [22]. Like overweight rates, hypertension is more prevalent in the formal neighborhoods. Hypertension also is more prevalent among those 50 and over, not married (e.g. widowed or divorced), individuals with a lower educational level, the economically inactive or self-employed people, Muslims, and migrants.

The multivariate analyses confirm and refine these figures (Table 6). After controlling for socio-economic characteristics of the respondents, residence in the formal versus informal neighborhoods no longer significantly influences the NCD behaviors, with the exception of overweight. Controlling for other variables, living in a formal area, being female, middle-aged, married, well-off and educated, are all related to excessive weight. Difference in age structure in the two neighborhoods is the single most powerful factor underpinning the correlation of the NCD behaviors to neighborhood. When standardized age is used in the regressions the association between neighborhood and each NCD behavior is eliminated (except for overweight). Standardized age consistently influences the NCD behaviors. Being younger increases the propensity to smoke, while being older increases the likelihood of heavy drinking, having a relatively sedentary lifestyle, and having high blood pressure. Being male and Christian is associated with both smoking and drinking. The lower level of physical exercise among adults living in formal areas is related to differences in the level of poverty between the two areas. In addition, being female, non-poor, more educated and not working outside the home are all independently linked to lower levels of physical activity. When controlling for other factors, educational attainment is no longer associated with hypertension. On the other hand, not being in a union, born outside Ouaga, or Muslim are all independently linked to hypertension.

**Table 6:** Adjust odds ratios of predicators of NCDs, Ouagadougou HDSS, Health Survey 2010.

	Tobacco use (1 = current smoker)	Alcohol consumption (1 = heavy drinker)	Physical inactivity (1 = low level)	Overweight (1 = BMI> = 25)	Hypertension (1 = high blood pressure)
**Neighborhood**					
Informal	1.0 (0.5–2.0)	0.6 (0.3–1.2)	0.8 (0.6–1.1)	**0.4 (0.3–0.6)**	0.9 (0.6–1.3)
**Sex**					
Female	**0.0 (0.0–0.1)**	**0.4 (0.2–0.9)**	1.3 (0.9–1.9)	**2.4 (1.6–3.5)**	1.1 (0.8–1.7)
**AgeStd**	**0.5 (0.3–0.8)**	**1.8 (1.2–2.6)**	**1.2 (1.0–1.5)**	**0.8 (0.6–0.9)**	**1.8 (1.5–2.3)**
**Marital status**					
Not in union	0.6 (0.2–2.2)	1.1 (0.5–2.6)	1.0 (0.7–1.6)	**0.6 (0.3–0.9)**	1.3 (0.8–2.1)
**Standard of living**					
Middle	0.7 (0.4–1.3)	0.7 (0.4–1.3)	**1.4 (1.0–1.9)**	1.4 (0.9–2.1)	1.1 (0.7–1.6)
Rich	0.4 (0.1–2.6)	0.3 (0.1–1.9)	**2.4 (1.2–4.6)**	**2.6 (1.4–4.7)**	0.7 (0.3–1.4)
**Level of education**					
Primary	1.6 (0.8–3.2)	1.1 (0.5–2.6)	1.3 (0.9–2.1)	1.4 (0.8–2.3)	1.3 (0.8–2.2)
Secondary or more	1.4 (0.5–3.5)	1.1 (0.3–3.6)	1.7 (0.9–3.0)	**2.1 (1.2–3.8)**	1.3 (0.6–2.6)
**Occupation**					
Employee	0.7 (0.2–2.1)	0.6 (0.2–1.8)	**0.5 (0.3–0.8)**	0.9 (0.5–1.6)	0.6 (0.3–1.1)
Independent	1.0 (0.4–2.5)	0.9 (0.5–1.8)	**0.5 (0.3–0.7)**	1.2 (0.8–1.7)	1.0 (0.7–1.4)
**Ethnicity**					
Not Mossi	0.8 (0.2–3.2)	1.2 (0.3–4.2)	1.2 (0.7–2.1)	1.4 (0.8–2.4)	1.2 (0.6–2.2)
**Religion**					
Muslim	**0.5 (0.3–1.0)**	**0.0 (0.0–0.1)**	1.0 (0.7–1.4)	1.0 (0.7–1.4)	**1.5 (1.0–2.1)**
**Place of birth**					
Outside Ouaga	0.9 (0.3–2.1)	0.9 (0.4–2.0)	1.1 (0.7–1.6)	1.0 (0.6–1.4)	**1.5 (1.0–2.4)**
**Log Likelihood**	−177.305	−221.019	−662.808	−542.953	−599.559

Notes: Odds Ratios are adjusted for all other variables in the model. For reference groups for the independent variables, see Table 5. Bold indicates significant differences across variable categories. The parentheses indicate a 95% confidence interval.

## Discussion

Our study reveals a high level of premature mortality due to NCDs in the peripheral neighborhoods of Ouagadougou. While mortality rates attributable to NCDs grow rapidly after age 50 in both formal and informal neighborhood, they increase more rapidly in formal areas. After age 50, the NCD death rates in the formal neighborhoods are double the rates found in the informal neighborhoods. The mortality rates due to communicable disease mortality rates also remain high among adults, especially in informal areas; HIV AIDS accounts only partially for this excess adult communicable disease mortality. The higher rates of mortality due to NCDs in the formal neighborhoods may reflect the long-term impact of some of the socio-economic and lifestyle differences between the two neighborhoods. However, poverty and lower educational level are associated with higher risks of dying from both NCD and communicable diseases across neighborhood areas, which suggests that the excess mortality in formal areas is not only due to a compositional or a life style effect. Rather, the relatively low rate of mortality by NCDs in informal areas may reflect the custom for migrants to return home to their villages to get care and support when they have a chronic condition, especially for migrants who live in informal areas where households are typically small and lack the resources to care for chronically ill members [23].

NCD mortality is substantial by age 50. This suggests the need for prevention programs starting at relatively young ages in both types of neighborhoods, consistent with studies underscoring the need to begin prevention of diabetes risk factors, such as overweight and low levels of physical activity, among children and adolescents [24]–[25]. However, the current imbalance in causes of death between these two types of neighborhoods indicates that public health interventions need to be carefully designed to place reduction of NCD risk in the context of continuing communicable disease risk, particularly in the informal settlements [26]–[30].

Our study reveals a high prevalence of risk factors for NCDs in the studied neighborhoods, with over one-fourth of the adults being overweight and over one-fourth having hypertension. While we expected poorer chronic disease outcomes among the poor, as is the case in other contexts [25], [31]–[34], the evidence suggests that NCD risk factors touch all socio-economic subgroups in Ouagadougou. In sum, better off residents (wealthy or educated) are more prone to physical inactivity and excessive weight, while vulnerable populations such as widows/divorced individuals and migrants, suffer more from higher blood pressure. The associations reflect the patterns of higher obesity among wealthier individuals in previous studies conducted in Ouagadougou [21], [22], as well as the lack of relation between typical socioeconomic indicators and hypertension risk [23]. The higher blood pressure of Muslims could be explained by greater levels of social vulnerability. Christian churches typically offer strong social support networks in these newly settled neighborhoods. These results underscore the importance of carefully guiding programmatic interventions to increase healthy lifestyles, to ensure that they engage all socio-economic strata in the communities, rich and poor, educated and less educated, better and less socially and economically integrated. While both wealthier and more vulnerable adults are at a high risk of NCD in some respect, we should underline that NCD mortality is higher among poorer residents and residents with less schooling, showing the impact of differential access to treatment.

The remaining differences in NCD risk factors are important as well. Gender has a pervasive influence on NCD behavioral risk factors, but it varies by behavior. Females have a significantly lower risk of being smokers or heavy drinkers, while they are more likely to be physically inactive or overweight. While it is often found that marriage has a health protective effect, our results show that in the study population, marital status is not associated with any of the risk factors except for overweight, where those who are not in a union are less likely to be overweight. The combination of these two factors together suggests that married women are particularly at risk for becoming overweight. Creative strategies are needed to reach these women, including through activities which come right to their doorstep or reach into the social networks. Another analysis of the same data showed that among the non-married, only non-married *women* (widows and divorcees) have a greater risk of hypertension [35]. Muslim individuals are less likely to be smokers or heavy drinkers, suggesting the importance of faith-based groups as entry-points into the community for NCD prevention programs.

In the formal neighborhoods, we found a higher prevalence of sedentary lifestyles (74%), overweight (38%) and hypertension (33%). Though uncommon, only smoking was more common in the informal neighborhoods (about 11%). After controlling for the simultaneous influence of all other variables in the model, whether someone lives in a formal versus informal neighborhood has no significant association with behaviors contributing to NCD risk, except for overweight, where living in an informal neighborhood reduces overweight risk. The formal neighborhoods have a greater density and access to stores, markets, street food, restaurants and bars. Are more of the households in these neighborhoods purchasing and consuming more of the high-density foods typically sold by these vendors? Walking and physical exercise is also less frequent in the formal neighborhoods. Is the greater prevalence of overweight among residents of formal areas also related to their greater access to vehicular transport, to their greater proximity to the center and their work place? While additional research is needed to clarify the exact nature of differences in consumption and exercise patterns across neighborhoods, it is clear that interventions are needed to overcome the factors pushing more adults to become overweight in the formal neighborhoods.

These results should be considered with several limitations in mind. First, the ages of the older individuals followed by the Ouaga HDSS are often estimated, given that ID cards frequently mention only an approximated year of birth (“né vers.”). However, by working with age groups instead of exact ages, we are able to overcome this weakness to some extent. Another limitation is that the cause of about one in five deaths remains unknown or ill-defined, because we were not able to collect data on these deaths (e.g., the family had moved) or, because the data was not sufficient to determine a cause. In this analysis, we proportionally redistributed these deaths from ill-defined or unknown cause. Though assessing a cause to a death is probably not independent of the cause itself, this is the most common way to consider these deaths in absence of more detailed information. Finally, while a majority of the population do not move between data collection rounds, in-migration and out-migration remain important in those peripheral neighborhoods of Ouagadougou, and even more in the informal areas [12]. Selective out-migration in informal areas probably leads to underestimated mortality levels, especially the one due to NCDs.

### Conclusions

The burden of NCD mortality touches adults at a relatively early age in African cities, regardless of their income or residence. The analyses presented in this study highlight the need to work with both poorer and wealthier groups on NCD prevention in Ouagadougou, all socio-economic groups being concerned, albeit by different diseases and behaviors. Health care for NCDs remains rare and expensive in Ouagadougou, putting a significant financial burden on the wealthiest, and remaining inaccessible to the poor. In this context, prevention programs are a cost-effective and immediate strategy to reduce the level of NCD and delay their onset. A number of programs have been shown to be effective in low-income groups in richer nations, and in particular programs involving a community, social-support dimension [24], [36], [37]. These programs now need to be adapted to the urban sub-Saharan African context.

While further studies are needed to clarify the specific dietary and specific physical activity differentials associated with the NCD risks documented in this study, the data presented make clear the pressing need to develop effective programs to reduce NCD risk across all types of neighborhoods in African cities. Tailored programs are needed. The analysis points in particular to programs targeted at married women and towards various faith-based groups.
